# Characterization of Circulating Low-Density Neutrophils Intrinsic Properties in Healthy and Asthmatic Horses

**DOI:** 10.1038/s41598-017-08089-5

**Published:** 2017-08-10

**Authors:** Nicolas Herteman, Amandine Vargas, Jean-Pierre Lavoie

**Affiliations:** Department of Clinical Sciences, Faculty of Veterinary Medicine, Université de Montréal. 3200 Rue Sicotte, Saint-Hyacinthe, J2S 2M2 QC Canada

## Abstract

Low-density neutrophils (LDNs) are a subset of neutrophils first described in the bloodstream upon pathological conditions, and recently, in the blood of healthy humans. LDNs may have an enhanced pro-inflammatory (low-density granulocytes, LDGs) or an immunosuppressive (Granulocytic myeloid-derived suppressor cells, G-MDSCs) profile. Whether these characteristics are specific to LDNs or related to disease states is unknown. Thus, we sought to investigate the properties of LDNs in both health and disease states, and to compare them to those of autologous normal-density neutrophils (NDNs). We studied 8 horses with severe equine asthma and 11 healthy animals. LDNs were smaller and contained more N-formylmethionine-leucyl-phenylalanine receptors than NDNs, but the myeloperoxidase content was similar in both cell populations. They also had an increased capacity to produce neutrophil extracellular traps, and were more sensitive to activation by phorbol-12-myristate-13-acetate. This profile is suggestive of LDGs. These characteristics were similar in both healthy and diseased animals, indicating that these are intrinsic properties of LDNs. Furthermore, these results suggest that LDNs represent a population of primed and predominantly mature cells. This study is the first to characterize LDNs in health, and to compare their properties with those of NDNs and of animals with a naturally occurring disease.

## Introduction

Neutrophils are key players in the inflammatory response, and they are the first leukocytes to reach tissues to fight against infectious agents and various other aggressors^1^. They were initially considered terminally differentiated cells^2^, but it is now recognized that neutrophils are a heterogeneous cell population, composed of subsets displaying distinct properties^3, 4^. Low-density neutrophils (LDNs) are neutrophils that co-segregate with blood mononuclear cells after density-gradient separation techniques. They have been reported to be present in the bloodstream of human patients suffering from autoimmune disorders (e.g. systemic lupus erythematosus or SLE)^5–9^, cancer^10, 11^, systemic and local infection^12–17^, dermatomyelosis^18^, malaria^19^ and asthma^20^. LDNs have also been observed in the peripheral blood of pigs after experimental viral infection^21^ and of rats with pristane-induced arthritis^22^, but have not yet been reported in the blood of animals during naturally occurring disease processes.

Several findings in humans suggested that LDNs can display an enhanced pro-inflammatory profile with an increased synthesis of cytokines (TNF-α, IL-6/-8, IFN type I)^8^, capable of contributing to neutrophilic recruitment and persistence in chronic inflammatory conditions. They also have an increased proclivity to spontaneously produce neutrophil extracellular traps (NETs)^5^, in a process known as NETosis^23^, and circulating LDN levels are correlated with disease state and severity in humans^14, 20, 24^. Because of these findings, LDNs were initially considered as an aberrant, pathological population of cells^8, 24^, and the term “low-density granulocytes” (LDGs) has been introduced to describe LDNs with pro-inflammatory properties. Since then, however, LDNs were reported to also be present in the bloodstream of healthy human subjects^14, 20, 25^ suggesting that similarly to NDNs, they are a normal cell population that may be increased in number under disease conditions.

Because of their expression of defensin^9^ and their morphology^7^, it has been postulated that LDNs are immature cells, progenitors of normal-density neutrophils (NDNs) that are prematurely released from the bone marrow secondary to an increased recruitment during inflammation^26^. Conversely, it has been suggested that LDNs are mature NDNs activated following inflammatory signals^26^ or that they derived from progenitor cells distinct from those leading to NDNs^6^. However, it was also proposed that they were a mixed population^8^ or even, mostly mature cells^12^. Clearly, the presence of LDNs in the blood of healthy individuals, their maturation status, their origin, and their enhanced pro-inflammatory profile compared to NDNs, remain controversial. Therefore, the present study was performed to evaluate the properties of LDNs in health and during chronic asthmatic inflammation. We hypothesized that LDNs have several characteristics that may not be influenced by the health status of the subject. We first determined that LDNs were present in the blood of healthy horses and of animals affected with severe equine asthma (heaves), a neutrophilic inflammatory airway disease commonly affecting adult horses^27^. We then characterized these cells (morphologically, phenotypically and functionally) both in healthy and diseased horses.

## Results

### Quantification of Low-Density Neutrophils in the PBMCs

**Figure 1:**
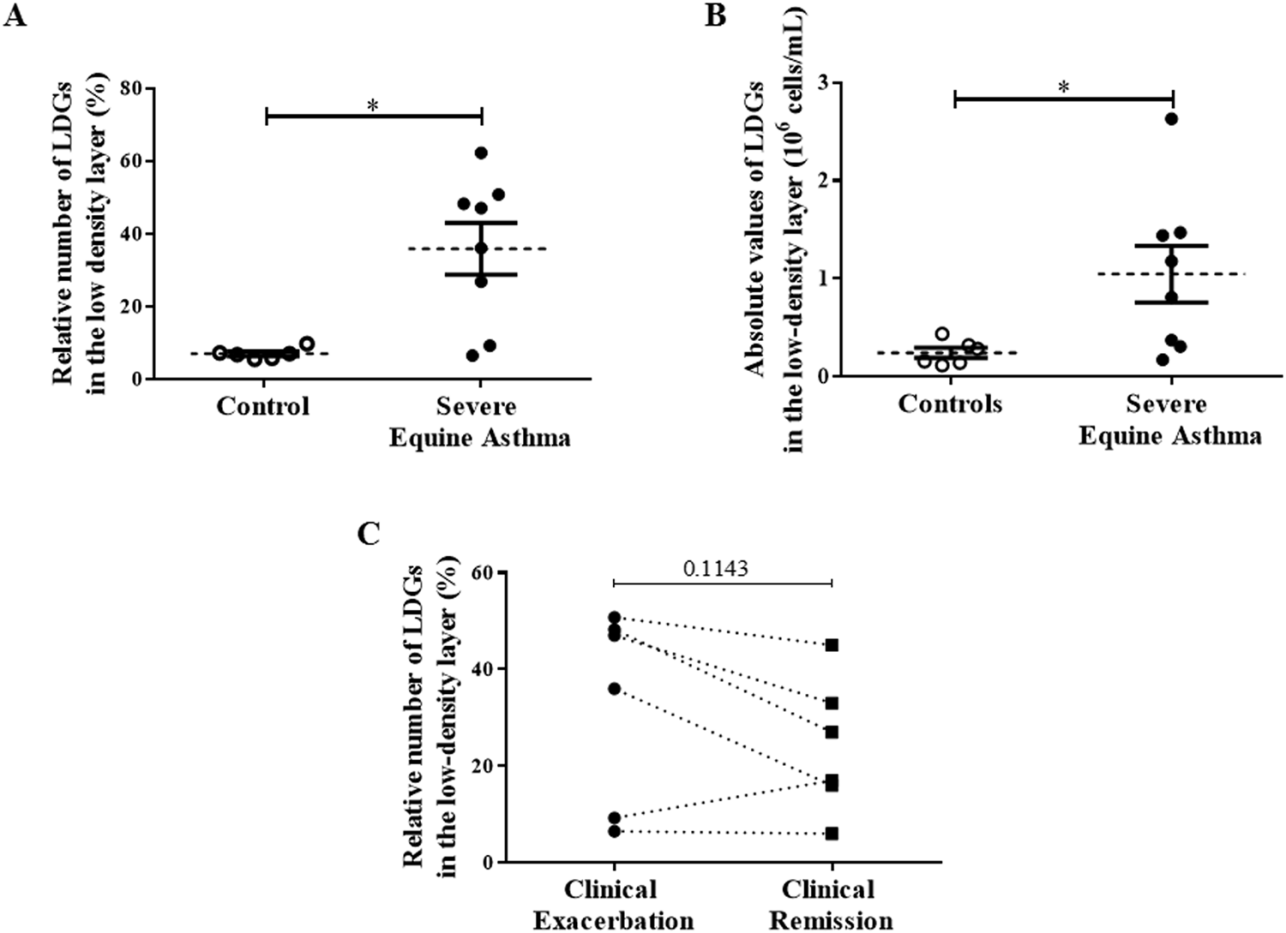
Levels of low-density neutrophils (LDNs). (**A,B**) Percentages and numbers of LDNs in peripheral blood mononuclear cells (PBMCs) of controls and horses with severe equine asthma during disease exacerbation. Each symbol denotes a single animal, and the mean ± SEM for each study population is shown. *p < 0.05 compared with control. (**C**) Percentages of LDNs in PBMCs of horses with severe equine asthma, comparing clinical exacerbation with clinical remission (p = 0.1143). Each symbol denotes a single animal.

LDNs were identified in the peripheral blood mononuclear cell (PBMC) layer from both healthy and asthmatic horses, and levels were not affected by the age and the sex of the animals. Horses with severe asthma in exacerbation of the disease had a significantly greater percentage (35.9% ± 7.13) and absolute number (1.05 × 10^6^ ± 2.89 × 10^5^ cells per ml) of LDNs in the PBMC layer compared with controls (7.0% ± 0.62, p = 0.05 and 2.48 × 10^5^ ± 5.19 × 10^4^ cells per ml, p = 0.03, respectively; Fig. 1A,B). The percentages of LDNs decreased during disease remission in 5 of the 6 asthmatic horses when compared to disease exacerbation, but this difference was not statistically significant (p = 0.11, Fig. 1C).

There was a significant increase of the NDN absolute number in severe equine asthma (3.94 × 10^6^ ± 5.91 × 10^5^) when compared to controls (2.63 × 10^6^ ± 3.05 × 10^5^, p = 0.04), but the values remained within the normal range for this species^28^. There were no other significant differences in the numbers of cells isolated from each layer in all groups (data not shown). Eosinophils were only found in the normal density layer. The values remained within the normal range for horses^28^ and no difference between control and asthmatic horses (4.73 × 10^5^ ± 1.13 × 10^5^ and 5.34 × 10^5^ ± 1.10 × 10^5^, respectively; data not shown) were observed.

### Morphological evaluation

**Figure 2:**
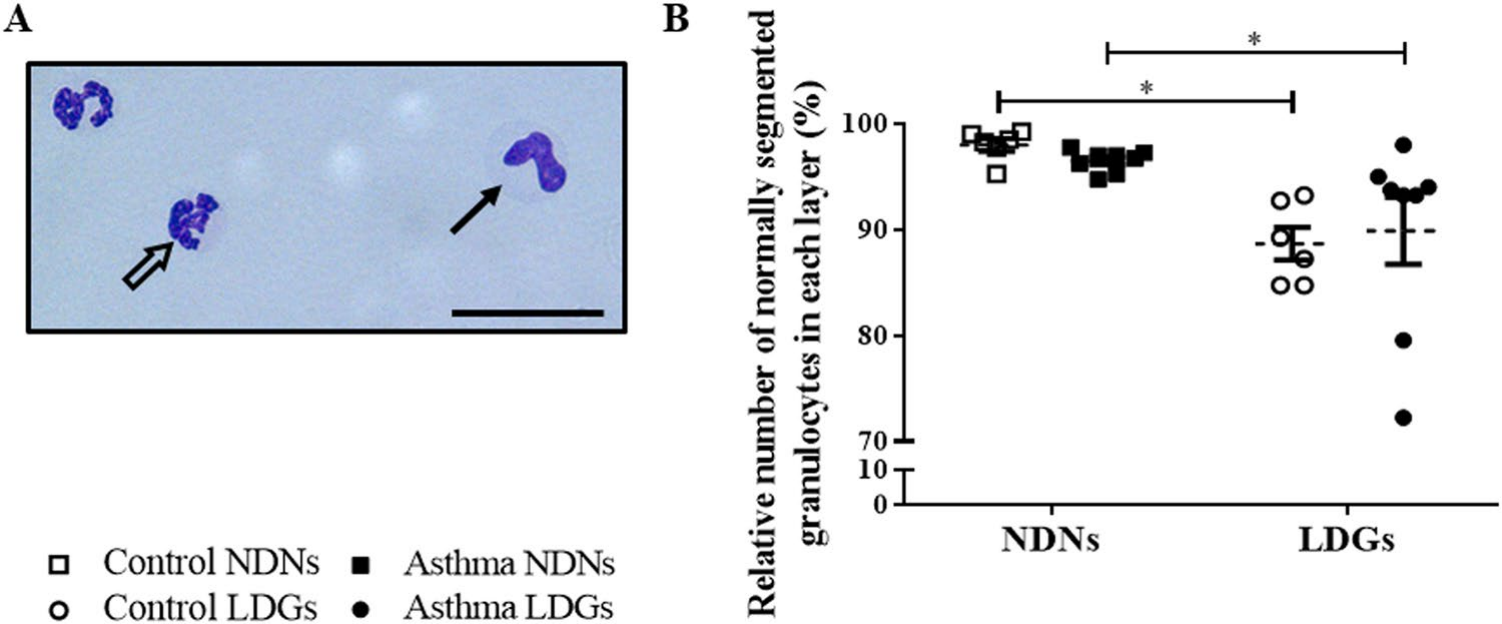
Levels of normally segmented granulocytes in each layer. (**A**) Representative photography of cytospins (x400, stained Protocol Hema 3) of the peripheral blood mononuclear cell layers (scale bar = 28 µm). Mature granulocytes (empty arrow) have more than 2 nuclear lobes (classically between 3 and 4) connected by filaments, whereas immature granulocytes (full arrow) have a curved nucleus with 2 or fewer nuclear lobes. LDNs were quantitated morphologically by light microscope. (**B**) Percentages of normally segmented LDNs in peripheral blood mononuclear cells of controls and horses with severe asthma. Each symbol denotes a single animal (mean ± SEM). *p < 0.05 compared with control.

Morphological evaluation was performed to assess the maturity of LDNs. Immature granulocytes were considered as having a hyposegmented nucleus with 2 lobes or less, but also a greater diameter (Fig. 2A)^29, 30^. In each group of horses, there was had significantly less LDNs with a normally segmented nucleus (88.7% ± 2.93 in control horses and 89.9% ± 2.53 in asthmatic horses) compared to NDNs (98.0% ± 0.59 in control horses, p = 0.03 and 96.5% ± 0.36 in asthmatic horses, p = 0.05; Fig. 2B).

**Figure 3:**
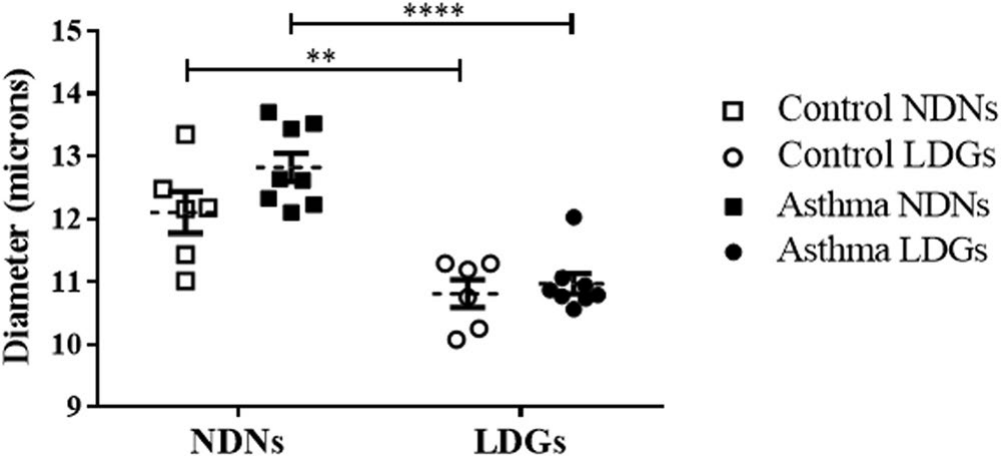
Size of Low-Density Neutrophils (LDNs) and Normal-Density Neutrophils (NDNs). Each symbol denotes the size (diameter) mean for a single horse, and the mean ± SEM for each studied population is shown. **p ≤ 0.01 and ****p ≤ 0.0001.

In both control and asthmatic horses, LDNs were significantly smaller (10.82 µm ± 0.22, and 10.98 ± 0.16 µm, respectively; Fig. 3) than NDNs (12.10 µm ± 0.34, p = 0.006 and 12.82 ± 0.22 µm, p < 0.0001, respectively). There was no significant effect of the condition on the segmentation of the nucleus, nor on the cell diameter in either type of granulocytes. However, there was a trend for NDNs from asthmatic horses to be bigger than those of controls (p = 0.08).

### Flow cytometry

**Figure 4:**
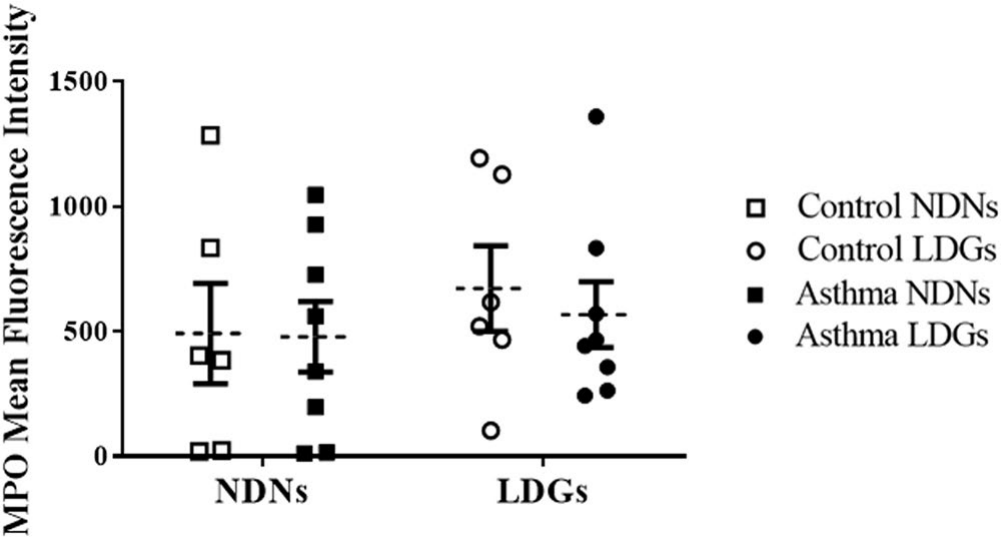
Mean Fluorescence Intensity of MPO in both layers of cells. Each symbol denotes the size mean for a single animal. The mean ± SEM for each studied population is shown.

The intracellular levels of myeloperoxidase (MPO) have been used to evaluate the maturity of neutrophils and to determine if the cells had degranulated^8^. LDNs and NDNs displayed comparable levels of intracellular MPO expression (Fig. 4) in the present study and it was not affected by the health status of the animals.

### Immunofluorescence

**Figure 5:**
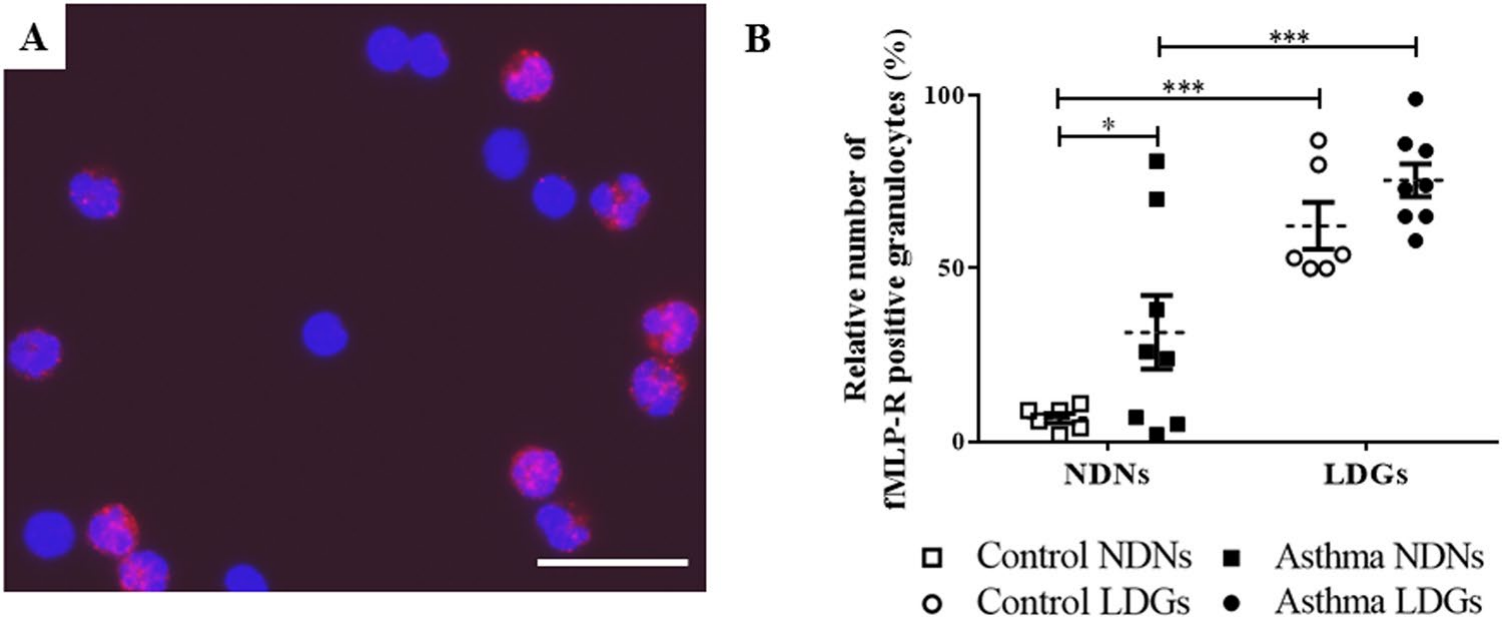
Immunofluorescence of fMLP-R in both layers of cells. (**A**) Representative photography at ×400 magnification (scale bar = 28 µm) of using an Axio Imager M.1 microscope (Zeiss) of the low-density layer. fMLP-R appeared as red points by immunofluorescence, giving the cells containing this receptor a piknotic aspect. The DNA appears in blue. (**B**) Percentages of low-density neutrophils and normal-density neutrophils positive for fMLP-R. Each symbol denotes a single animal. Mean ± SEM for each studied population is shown. *p ≤ 0.05 and ***p ≤ 0.001.

The expression of the N-formylmethionine-leucyl-phenylalanine receptor (fMLP-R) in the granules was measured as a marker for neutrophil maturity, as it has been shown to increases in mature in neutrophils^9, 31^. In this study, fMLP-R signal appeared as red at the immunofluorescence and the lobularity of the nuclei was used in order to identify the granulocytes (Fig. 5A). Significantly more LDNs expressed the fMLP-R (69.9% ± 4.22) when compared to NDNs (21.0% ± 6.79, p < 0.0001; Fig. 5B). While severe equine asthma had no impact on the expression of fMLP-R in LDNs, they were significantly increased in asthmatic NDNs compared to those from healthy horses (31.6% ± 10.6 and 6.83% ± 1.40, respectively; p = 0.05; Fig. 5B).

### NET production

**Figure 6:**
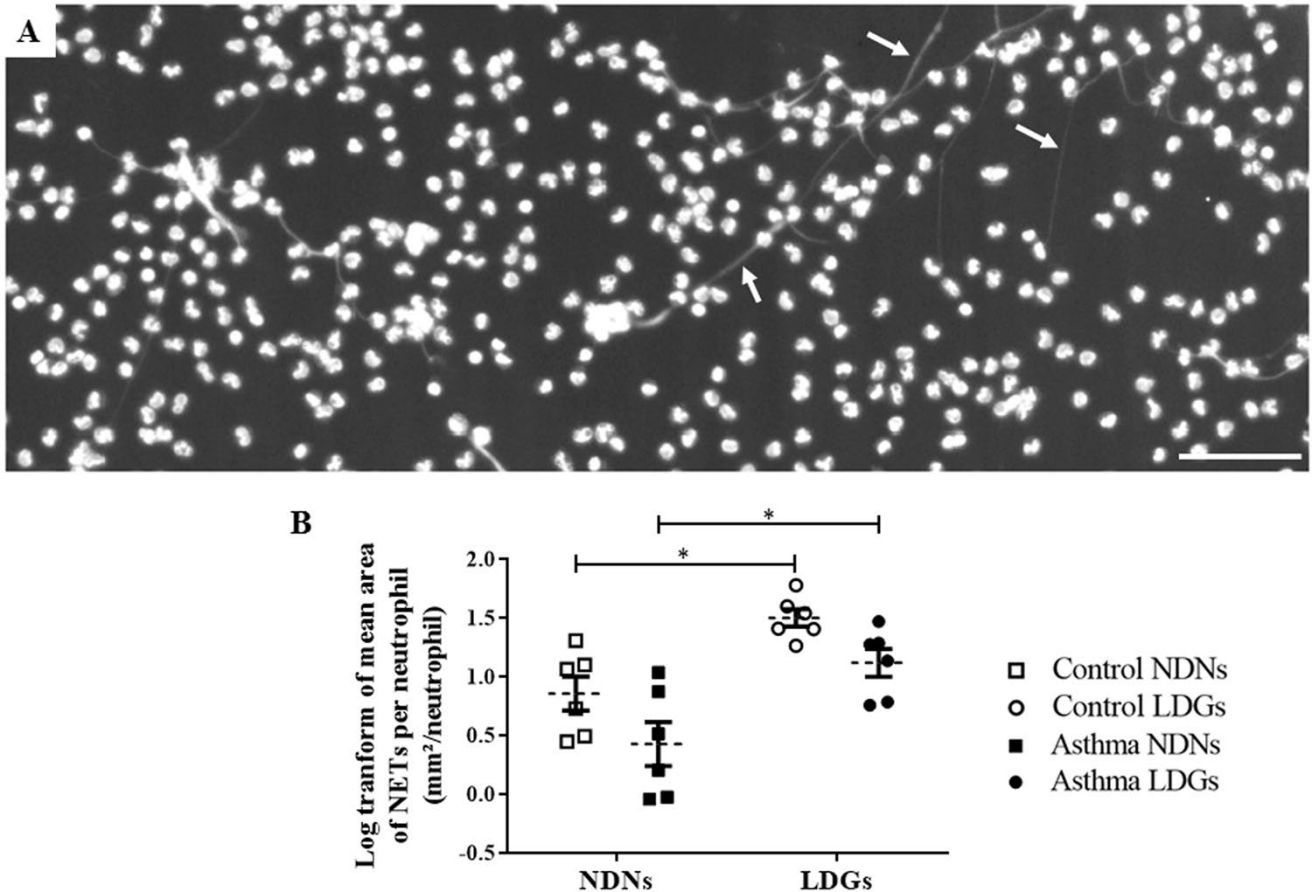
Neutrophil extracellular traps (NETs) production in both layers of cells. (**A**) Representative photography of using a MRC1024 confocal laser-scanning microscope at ×100 magnification (BioRad, Hercules, CA) equipped with a Nikon Eclipse TE300 camera (Nikon, Tokyo, Japan) of the low-density layer (scale bar = 100 µm). White arrows indicate NETs’ structures. (**B**) Log transform of the mean area of NETs per neutrophil in each layer (non-stimulated NS). Each symbol denotes a single animal. Mean ± SEM for each studied population is shown. *p ≤ 0.05.

NETs on confocal microscopy appear as a thin white filament originating from a nucleus and orientating toward another one (Fig. 6A). Spontaneous NET formation was enhanced in LDNs when compared to NDNs in both group of horses (Fig. 6B). After logarithm transformation, the mean NET area per neutrophil in control horses for LDNs and NDNs was −4.50 ± 0.07 log of µm²/neutrophil and −5.14 ± 0.14 (p = 0.03), respectively. In asthmatic horses, the values for LDNs and NDNs were −4.88 ± 0.12 and −5.57 ± 0.19, respectively (p = 0.02).

**Figure 7:**
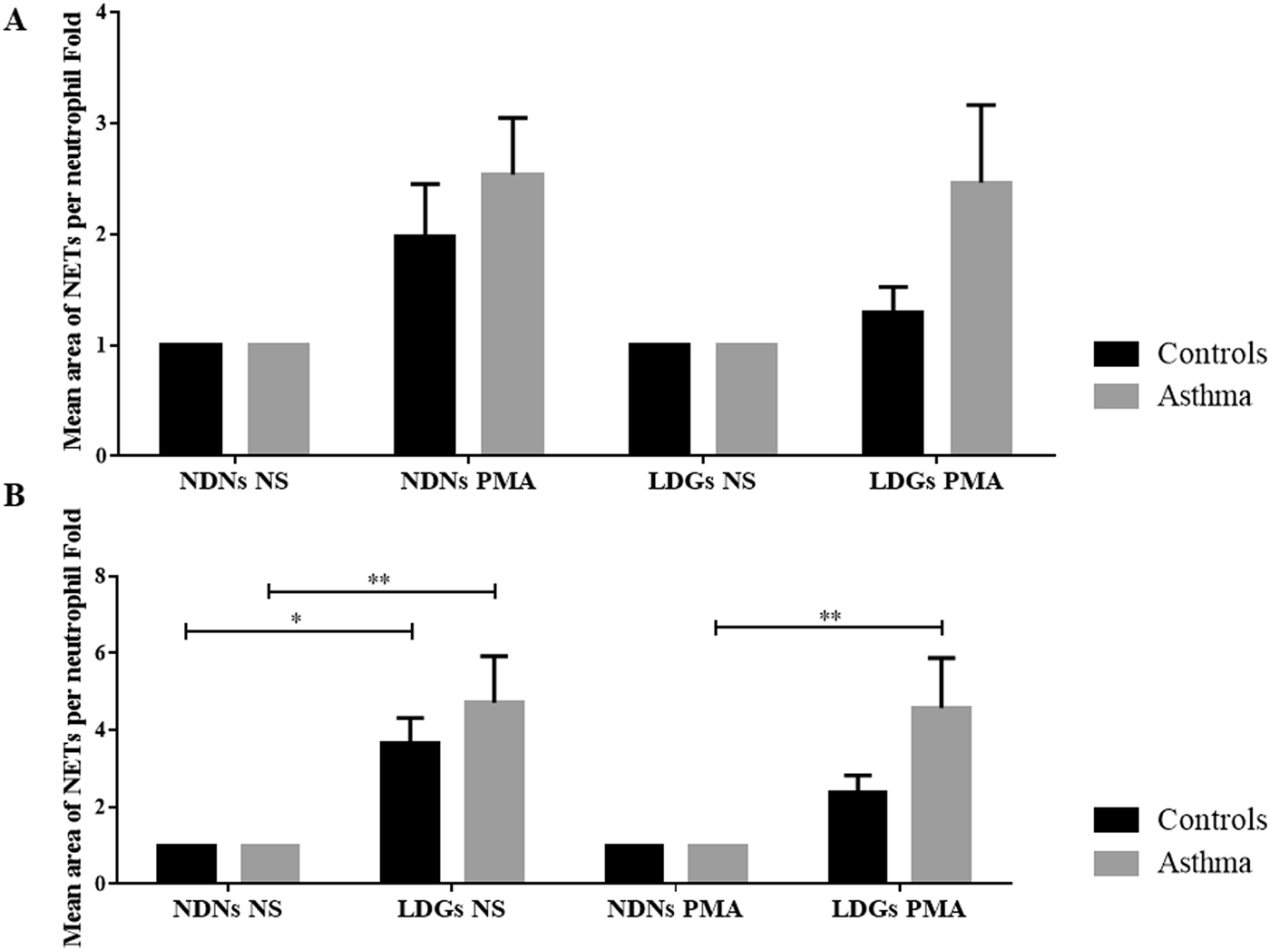
Fold changes of mean area of NETs per neutrophil in each layer of cells. (**A**) Results represent fold induction of the effects of PMA stimulation on the mean area of NETs per neutrophil on each layer of cells in each group of horses. Stimulation with PMA significantly increased the production of NETs in both groups of horses (p = 0.0008). Mean ± SEM for each studied population is shown. A two-way repeated measures ANOVA without Sidak’s multiple comparison post-tests has been realized in this case. (**B**) Results represent fold induction of the mean area of NETs per neutrophil by LDNs compared to NDNs in each group of horses, before and after stimulation with PMA. LDNs produced significantly more NETs than NDNs (p < 0.001). Mean ± SEM for each studied population is shown. *p ≤ 0.05 and **p ≤ 0.01.

Stimulation with phorbol-12-myristate-13-acetate (PMA) significantly increased the production of NETs by NDNs and LDNs in both groups of horses (p = 0.0008, Fig. 7A). Fold increases also indicated that LDNs produced significantly more NETs than NDNs (between 2.40 to 4.70 more, p < 0.001, Fig. 7B). When expressed as LDN/NDN ratios, NET mean area per neutrophil was significantly increased only in non-stimulated LDNs of control horses (3.70-fold increase and p = 0.03 without PMA, against 2.40-fold increase and p = 0.56 with PMA). However, asthmatic LDNs produced significantly more NETs with and without PMA (4.70-fold increase and p = 0.001 for non-stimulated LDNs, against a 4.60-fold increase and p = 0.002 for stimulated LDNs).

## Discussion

LDNs are now recognized as a subset of neutrophils that may be found in the blood of human patients in association with disease severity in various inflammatory conditions. Whether the low buoyancy of these cells results from degranulation of NDNs, or from a distinct property or maturation process is unclear, as their contribution to disease processes. The present study provides new insights into several aspects of LDNs during asthmatic inflammation, but also importantly, in health. LDNs were present in the bloodstream of healthy horses and their levels were increased in asthmatic animals, as observed in humans^20^. The LDNs levels in the blood of asthmatic horses decreased during clinical remission of the disease, but remained above those of healthy controls. Moreover, compared to autologous NDNs, LDNs exhibited morphological, phenotypical and functional differences that were present in both healthy and asthmatic horses. These results suggest that LDNs have intrinsic properties that are neither influenced nor secondary to asthma, but that these cells increase in number and may be primed during inflammatory states.

### LDNs have intrinsic properties

Several differences between LDNs and NDNs were observed in the present study. LDNs were smaller than NDNs, which might contribute to their low buoyancy. Phenotypically, LDNs also had a different granular content (more fMLP-R) compared to NDNs and they had an increased capacity to produce NETs. These differences were present in both healthy and asthmatic horses, suggesting that these properties may be intrinsic to LDNs. fMLP-R are chemoattractant receptors that belong to the G protein-coupled receptor family^32^. When activated by N-formyl peptides such as N-formylmethionine-leucyl-phenylalanine (fMLP), they contribute to the physiological defense against bacterial infection and cell disruption^33^. This increased fMLP-R in LDNs suggest that these cells are more sensitive to activation stimuli and is in agreement with their proposed enhanced pro-inflammatory profile^8^ and anti-bacterial activities.

LDNs are present in the bloodstream of healthy horses but there was a mean 5-fold increase in numbers in the blood of asthmatic horses, as reported in human asthma^20^. The percentages of LDNs decreased, although not significantly, during disease remission when compared to exacerbation in asthmatic horses, and remained above values of controls, indicating that levels of LDNs vary with the severity of the disease. This is in agreement with the association between LDNs and human asthma severity^20^ and the possible role of the LDNs as a clinical biomarker. The lack of significant difference between these two disease states is likely explained by the low power of the study for this effect (it was estimated that 2 additional asthmatic horses would have been necessary in order to have 80% of chances to observe a significant difference). Also, a longer period of antigen avoidance (6 weeks in the present study) may have resulted in a significant decrease of the LDNs when compared to exacerbation state or even to a complete resolution of the asthmatic inflammation (LDN values similar to control horses). Indeed, in patients with pulmonary tuberculosis, LDN levels required 2 weeks of medical treatment to significantly decreased, and a 6-month period to be equal to those of healthy volunteers^12^.

### LDNs are a mixed population of immature and mature cells

LDNs were first considered as immature cells because of their low buoyancy^7, 9, 13^, their elevated expression of the cluster of differentiation 33 (CD33)^14^, and their granulopoiesis signature^9^. In the present study, the receptor for the chemoattractant fMLP was used to assess maturity as it is synthesized in the final stage of the maturation of the neutrophils^34^. The increased expression of fMLP-R in LDNs we observed at the proteomic level is in agreement with the increased mRNA expression found in SLE children^9^. Associated with the decreased diameter and the segmentation of the nuclei^30^, with the lack of difference of the MPO content we observed, these findings suggest that LDNs are mostly a mature population of neutrophils, unlike what was initially suggested^18, 26^. LDNs were also reported as mature neutrophils based on the surface molecular expression (CD10, CD15, CD16, CD66b, CD11b), although some cells had hyposegmented nuclei (band cells, lobular nuclei) rather indicating immaturity^8, 9, 20, 21^.

### NET production

Their increased formation of NETs in the present study suggests that LDNs in equine asthma have pro-inflammatory properties and may then belong to the group of LDGs. Furthermore, it suggest that they are more sensitive to activation stimuli compared to NDNs. NETs are chromatin filaments released by neutrophils that are associated with nuclear, cytoplasmic and granular proteins^23^. They have a function in host defense by protecting against pathogens and may cause direct epithelial and endothelial cell damages, by releasing toxic proteins (such as MPO)^35^ and by exposing autoantigens^36^. Two studies in humans^5, 37^ have also reported that unstimulated LDNs undergo significantly more NET formation compared to NDNs. In severe equine asthma, but not in healthy horses, unstimulated and stimulated LDNs produced almost the same amount of NETs and they were more sensitive to the stimulation compared to NDNs. This is in agreement with the results of Villanueva *et al*.^5^ in SLE patients, and support the hypothesis that these cells are primed in the diseased subjects^24^.

Several hypotheses regarding LDN origin have been suggested. An unidentified stimulus may alter the normal development of neutrophils in the bone marrow (e.g. early release, altered gene expression)^22, 26^ or may act directly on mature circulating neutrophils resulting in their lower buoyancy (e.g. activation, degranulation)^12, 26^. Another hypothesis proposes that LDNs and NDNs originate from different progenitor cells^6^. The evaluation of their surface markers in SLE patients indicated that LDNs may display some characteristics of activated neutrophils. However, other indexes (L-selectin shedding, levels of MPO and of ROS, transmission electron microscopy) rather suggested that they are not an activated and degranulated subset of neutrophils^8, 26^. Our results with MPO mean fluorescence intensity would be in agreement with this latter observation. However, the increased NETs formation and fMLP-R production suggest that LDGs may nevertheless be more easily activated than NDNs^30^. It is also consistent with their enhanced pro-inflammatory profiles in asthma as formylated peptides are well-known pro-inflammatory molecules^38, 39^.

### Horses and severe equine asthma as a model for LDN study

Severe equine asthma is a spontaneously and commonly occurring disease of adult horses, associated with bronchospasm, mucus accumulation and remodeling of the airways leading to periods of dyspnea^40^. Airway neutrophilia is a characteristic finding of this condition, with these cells infiltrating the lungs of susceptible horses as early as 5–6 h after antigen exposure, and preceding the development of airway obstruction^41^. The increased circulating LDNs we observed in asthmatic horses indicates that they possibly contribute to the disease expression, by enhanced NET production. The presence of NETs in the lungs of asthmatic horses but not in controls has been reported^42^.

Equine asthma is not only a disease of veterinary importance, but it is also considered as a suitable model for human asthma^27, 40^, because of the numerous similarities between the conditions. Furthermore, equine and human neutrophils have similar biology^30, 43–46^ and the remodeling of asthmatic airways affects the epithelium, extracellular matrix and smooth muscle layers in both species^40^. Results of the current study also indicate that horses are an appropriate model to study unresolved issues regarding the origin or the pathophysiology of LDNs in health, and their contribution to neutrophilic asthma. The size of horses facilitates these studies, as it allows collecting non-invasively large amount of blood (and cells) without altering the animal immune response and measuring various physiological parameters (bronchoalveolar lavage fluid or BALF, respiratory mechanics, and lungs biopsies), without anesthesia, or scarifying the animals, as in rodents.

In conclusion, results of the present study suggest that LDNs in equine asthma are a population of mostly mature and primed cells, having characteristics that are distinct from those of NDNs, in both health and disease. According to the criteria enunciated by the Scapini *et al*.^3, 47^, it appears that LDNs in asthma have pro-inflammatory properties and are then LDGs. Our study also highlights the possible contribution of LDNs to domestic animal diseases and the suitability of horses as a model for the study of LDNs in human asthma. The presence of LDNs in healthy patients suggests that they could be a physiologic subset of neutrophils with a purpose in the homeostasis of the organism and that their increased expression in some disease cause a dysregulation contributing to the pathogenesis. However, more data are required before to assess this hypothesis.

## Material and Methods

### Experimental design

#### Study 1

14 horses (6 healthy and 8 asthmatic) were stabled and fed hay for at least 30 days to cause exacerbation of asthma in susceptible animals. The amount of circulating LDNs, and the morphological (diameter and segmentation of nuclei) and phenotypical (flow cytometry and immunofluorescence) evaluations of neutrophils (LDNs and NDNs) were studied. The amount of circulating LDNs was also evaluated in 6 of these asthmatic horses while at pasture for 6 weeks to induce clinical remission of the disease.

#### Study 2

The production of NETs by LDNs and NDNs was assessed in 12 horses (6 healthy and 6 asthmatic) stabled and fed hay for at least 30 days.

### Animals

Eight mixed-breed adult horses with severe asthma (means of 527.6 ± 16.3 kg and 15.1 ± 1.78 years of age, mean ± SEM) and 11 age-matched healthy controls (means of 512.7 ± 7.41 kg and 12.4 ± 1.16 years of age) from the research herd of the Equine Asthma Research Laboratory at the Université de Montréal (including 16 mares and 3 geldings) were studied. The two groups of horses were housed together during the entire course of the study. Horses with severe equine asthma had a previous history of airway obstruction documented by lung function measurements and pulmonary neutrophilia in BALF (≥25%) upon stabling and hay feeding^48^. Control horses had no history or clinical signs suggesting airway diseases. The degree of respiratory impairment in horses were assessed daily by clinical scoring^49, 50^. A score from 0 to 4 is attributed to nasal flaring (0: no flaring; 4: severe, continuous flaring during each respiration) and abdominal movement (0: no abdominal effort; 4: severe, marked abdominal movement). Both scores are added for a maximal score of 8. Scores ≥4 indicates respiratory dysfunction. Furthermore, at the beginning of the study and at the time of the sampling, respiratory mechanics were performed using an impulse oscillometry (IOS) device as described by Van Erck *et al*.^51^ with the Equine MasterScreen IOS system (Jaeger, Würzburg, Germany). However, these data are not presented in this paper because part of another study conducted by Fillion-Bertrand *et al*. (paper submitted) at the same time than our and including the same horses. All experimental procedures were performed in accordance with the guidelines of the Canadian Council for Animal Care and were approved by the Animal Care Committee of the Faculty of Veterinary Medicine of the Université de Montréal (Rech-1716).

### Neutrophil isolation

**Figure 8:**
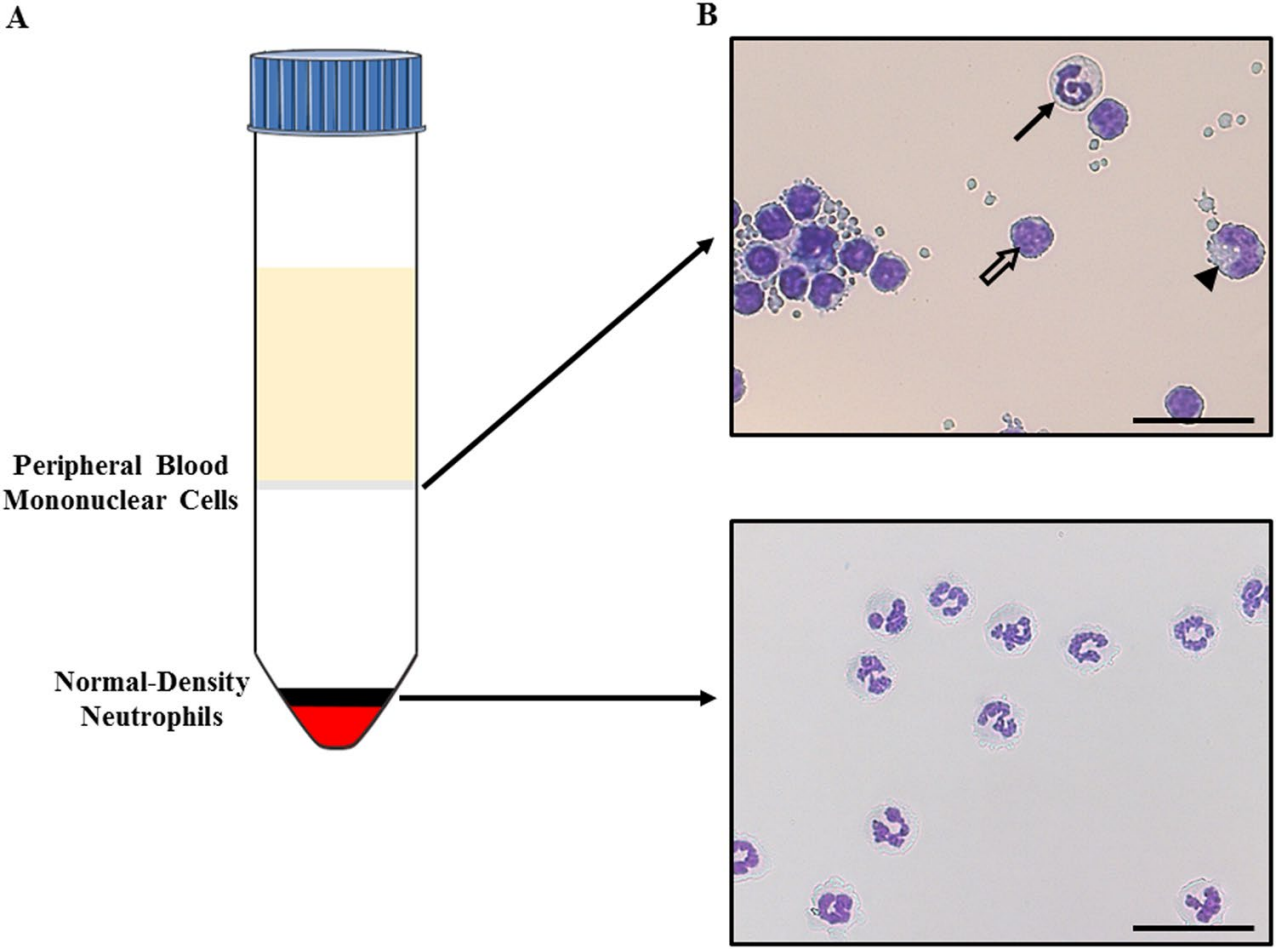
Isolation procedure for the neutrophil subsets from equine blood. (**A**) Ficoll density gradient separating Low-Density Neutrophils (migrating at the interface blood/gradient together with peripheral mononuclear cells) from Normal-Density Neutrophils migrating at the bottom of the gradient together with red blood cells. (**B**) Neutrophil subsets morphology evaluated by optical microscopy at 400× magnification (scale bar = 28 µm). Upper panel: LDNs (full arrow) with mononuclear cells (lymphocytes indicated by the empty arrow and monocytes indicated by the arrowhead). Lower panel: NDNs after erythrolysis by a hypotonic treatment. Neutrophil subsets were cytocentrifuged and stained with Protocol Hema 3 and imaged using the NanoZoomer 2.0-HT system.

Blood was drawn by venipuncture in a jugular vein using sterile heparinized tubes (Tyco healthcare, Pointe-Claire, QC, Canada). NDNs and peripheral blood mononuclear cells (PBMCs) were isolated according to the manufacturer’s instructions. Briefly, after 30 to 45 minutes of sedimentation, the plasma-rich layer was recovered and used in a density gradient centrifugation method with Ficoll-Paque^TM^ Premium 1084 (GE Healthcare Bio-sciences Corp, Mississauga Canada). Five ml of the PBMCs layer was harvested (Fig. 8) and the NDN layer was collected in the bottom of the tubes after erythrocyte lysis using a hypotonic treatment with distilled water (Thermo Fisher Scientific, Burlington, ON, Canada). Cells were washed and suspended in a buffer solution containing phosphate-buffered saline (PBS) 1X, EDTA 0.5 mM (Thermo Fisher Scientific), and BSA 0.2% (Sigma-Aldrich, St Louis, MO, USA). Cell counting and viability were evaluated using ADAM automatic Cell Counter (Montreal-Biotech Inc., Montréal, QC, Canada). The viability of NDNs and PMBCs were 98.23 ± 0.22% (mean ± SEM), and 98.63 ± 0.33%, respectively.

Cytopreparations (Cytospin, Rottorfix Hettish) were stained with Protocol Hema 3 (Fisher Canada, Nepean, Canada) and a differential count performed on 400 cells, with the assessor blinded to sample origin. The purity of NDNs was 97.7% ± 0.37. There were 5.77% ± 0.58 LDGs in PMBCs layers for the control horses and 26.81% ± 4.19 for the asthmatic horses. Cells were then fixed 20 minutes in paraformaldehyde 2%, washed three times in PBS 1X and stored in 500 µL of PBS 1X at 4 °C until analyses.

### Flow cytometry

Intracellular MPO content was evaluated in each layer (NDNs and LDNs). Prior to staining, 10^6^ cells were harvested and washed twice in PBS 1X. All antibody incubation steps were performed at room temperature.

Granulocytes and PMBCs/LDNs were resuspended in blocking buffer (PBS 1X containing 2% FBS) and incubated on ice for 20 minutes. The cells suspension was then permeabilized with 0.3% Triton X-100 (Sigma-Aldrich) for 5 min and incubated with anti-rabbit MPO (IgG, 16 mg/L, Dako, Denmark) and with a monoclonal canine DH24A antibody^52–54^ (IgM, 15 µg/mL, VMRD, Pullman WA, USA) for 45 minutes in order to select equine neutrophils. After three washes in washing buffer, cells were incubated in dark for 45 min with secondary goat Alexa488-coupled anti-rabbit IgG antibodies (1:500 in washing buffer, Thermo Fisher Scientific) and goat PE anti-mouse IgM antibody (1:1000 in washing buffer, Invitrogen). Cells were then washed twice in washing buffer (PBS 1X) and suspended in 500 μL PBS before flow cytometry acquisition of 10 000 events and analysis using Cellquest Pro software on a FACScalibur instrument (BD Biosciences). Isotype-matched control antibodies (mouse IgM and rabbit IgG) were used to set photomultipliers (PTM) voltage and compensation parameters for fluorescence detection in FL-1 and FL-2 channels.

### Immunofluorescence

fMLP-R expression was evaluated in each layer (NDNs and LDNs). Prior to staining, 10^6^ cells were harvested and washed twice in PBS 1X.

Granulocytes were resuspended in blocking buffer (PBS 1X containing 2% FBS) and incubated on ice for 20 min. The cell suspension was then permeabilized with 0.3% Triton X-100 and incubated with anti-FPRL1 antibody [GM1D6] (2 mg/mL; ab26316, Abcam, Germany) for 45 min. Cells were washed three times in washing buffer and incubated in dark for 45 min with secondary goat Alexa594-coupled anti-mouse IgG antibody (1:500 in washing buffer, Invitrogen) and 50 μg/ml of 4,6-diamidino-2-phenylindole (DAPI, Vector Laboratories). Cells were then washed twice in washing buffer and suspended in 500 μL PBS 1X, for finally being mounted in a drop of ProLong Antifade reagent (P36930; Thermo Fisher Scientific).

Images were taken using an Axio Imager M.1 microscope (Zeiss) and analyzed using Zen software (Fig. 5A). A library of images was randomly established in order to have at least 200 granulocytes for each slide. Cells were identified as neutrophils based on nuclear morphology (segmentation of the nucleus) and because of the paucity in eosinophils and basophils (data not shown) by an operator blinded to horses and to the layer.

### Morphological evaluation

Granulocytes (NDNs and LDNs) were classified as immature or mature according to their nuclear segmentation. Nuclei displaying >2 nuclear lobes were considered as mature, those with ≤2 lobes were classified as immature granulocytes (Fig. 2A). At least 400 granulocytes were evaluated by an assessor blinded to sample origin.

All slides were then digitized at 200× magnification with the NanoZoomer 2.0-HT system (Hamamatsu Photonics, SZK, Japan). The diameter of each type of granulocyte was measured using ImageJ (http://rsb.info.nih.gov/ij/) with cells approximated as circles. The evaluator was blinded to slide identification and measurements were over 200 randomly selected granulocytes.

### Induction of NET formation and DNA staining

Neutrophils were isolated as described above except that blood was drawn in sterile EDTA tubes, as heparin dismantles NETs^55^. They were resuspended in complete RPMI and seeded (10^6^) onto six-well plates containing 1.5 mm-thick poly-L-lysine-coated coverslips, stimulated for 3 h with 200 nM PMA, and fixed for 20 min in cold methanol. After three washes in PBS 1X, a DNA-staining technique is performed by incubation of cells with propidium iodide (PI; 50 μg/ml) for 5 min at room temperature, and washed three times with PBS. The coverslips were then mounted in a drop of ProLong Antifade reagent (Thermo Fisher Scientific) and images were acquired with a MRC1024 confocal laser-scanning microscope at magnification ×100 (BioRad, Hercules, CA) equipped with a Nikon Eclipse TE300 camera (Nikon, Tokyo, Japan) and a Perfect Focus System (Fig. 7A).

### NET quantification

NET production was blindly assessed with NewCast software version 4.5.1.324b (Visiopharm, Denmark). A region of interest (ROI) was defined, for each image in order to exclude the border of the slides (5 mm from the border of each side). The regions where the focus prevented a reliable assessment of the cells were excluded. The NET mean area per granulocyte was assessed on 25% of the ROI (randomly selected by the software). A point counting technique using grids with 900 crosses per screen was performed (this point density allowed to reliably evaluate the structures of interest). NET mean area per granulocyte was calculated for each horse as follows: A_NETs_ = (4*A_cross_*ΣP_NETs_)/(Estimated number of granulocytes), where A_cross_ indicates the area occupied per one cross (area of the ROI divided per 900) and ΣP_NETs_ the sum of the points falling onto a NETs. The differential count of granulocytes per layer allowed the correction of the calculated area by the number of studied cells in each image (differential * 1 × 10^6^).

### Statistical analysis

Analyses were carried out using Prism 6.05 (GraphPad Software Inc, CA, USA). For cells quantification, data were analyzed with unpaired t-tests with Welch’s correction. For all other analysis, a two-way repeated measures ANOVA with a Sidak’s multiple comparisons post-test. For NET quantification, differences between means were analyzed using t test or by a two-way repeated measures ANOVA with or without Sidak’s multiple comparison post-tests, where a p ≤ 0.05 was considered significant.

All the results are expressed regarding the following presentation: mean ± SEM.

The datasets generated during and/or analysed during the current study are available from the corresponding author on reasonable request.
